# Cattle transhumance and agropastoral nomadic herding practices in Central Cameroon

**DOI:** 10.1186/s12917-018-1515-z

**Published:** 2018-07-03

**Authors:** Paolo Motta, Thibaud Porphyre, Saidou M. Hamman, Kenton L. Morgan, Victor Ngu Ngwa, Vincent N. Tanya, Eran Raizman, Ian G. Handel, Barend Mark Bronsvoort

**Affiliations:** 1 The Roslin Institute, Royal (Dick) School of Veterinary Studies, University of Edinburgh, Edinburgh, Easter Bush, Midlothian, EH25 9RG UK; 2 The European Commission for the Control of Foot-and-Mouth Disease (EuFMD) - Food and Agricolture Organization (FAO), Viale delle Terme di Caracalla, 00153 Rome, Italy; 3 Institute of Agricultural Research for Development, Regional Centre of Wakwa, Ngaoundere, P.O. Box 454, Cameroon; 4 Institute of Ageing and Chronic Disease and School of Veterinary Science, University of Liverpool, Leahurst Campus, Neston, Liverpool, Wirral CH64 7TE UK; 5 School of Veterinary Medicine and Sciences, University of Ngaoundere, Ngaoundere, P.O. Box 454, Cameroon; 6 Cameroon Academy of Sciences, Yaound’e, P.O. Box 1457, Cameroon; 7 Food and Agriculture Organization (FAO), Animal Production and Health Division, Viale delle Terme di Caracalla, 00153 Rome, Italy; 8 Royal (Dick) School of Veterinary Studies, University of Edinburgh, Easter Bush, Edinburgh, Midlothian EH25 9RG UK

**Keywords:** Transhumance, Cameroon, GPS, Cattle, Livestock movements

## Abstract

**Background:**

In sub-Saharan Africa, livestock transhumance represents a key adaptation strategy to environmental variability. In this context, seasonal livestock transhumance also plays an important role in driving the dynamics of multiple livestock infectious diseases. In Cameroon, cattle transhumance is a common practice during the dry season across all the main livestock production zones. Currently, the little recorded information of the migratory routes, grazing locations and nomadic herding practices adopted by pastoralists, limits our understanding of pastoral cattle movements in the country. GPS-tracking technology in combination with a questionnaire based-survey were used to study a limited pool of 10 cattle herds from the Adamawa Region of Cameroon during their seasonal migration, between October 2014 and May 2015. The data were used to analyse the trajectories and movement patterns, and to characterize the key animal health aspects related to this seasonal migration in Cameroon.

**Results:**

Several administrative Regions of the country were visited by the transhumant herds over more than 6 months. Herds travelled between 53 and 170 km to their transhumance grazing areas adopting different strategies, some travelling directly to their destination areas while others having multiple resting periods and grazing areas. Despite their limitations, these are among the first detailed data available on transhumance in Cameroon. These reports highlight key livestock health issues and the potential for multiple types of interactions between transhumant herds and other domestic and wild animals, as well as with the formal livestock trading system.

**Conclusion:**

Overall, these findings provide useful insights into transhumance patterns and into the related animal health implications recorded in Cameroon. This knowledge could better inform evidence-based approaches for designing infectious diseases surveillance and control measures and help driving further studies to improve the understanding of risks associated with livestock movements in the region.

**Electronic supplementary material:**

The online version of this article (10.1186/s12917-018-1515-z) contains supplementary material, which is available to authorized users.

## Background

In sub-Saharan Africa (SSA), transhumance of livestock is a common practice for pastoralist communities to cope with local environmental constraints, and fully exploit seasonal availabilities of grazing and water resources [1–3]. Transhumance, therefore, describes the movement of pastoralists and their livestock in response to the variability of environmental and ecological resources [1, 2]. Usually, these migrations are towards regions of different climate and tend to be to remoter riverine areas with poorer veterinary or medical facilities [2]. Long-distance livestock movements can contribute to the dissemination of endemic diseases, or to the introduction and spread of exotic animal diseases [4]. In particular, increased movements and mixing of stock during transhumance are common risks factors for the dissemination of a number of diseases in SSA [5, 6].

In Cameroon, transhumance is an established practice among cattle herders to overcome the constrains of the dry season [7, 8], which usually extends from September/October through to April/May of the following year [3]. During this period, a large proportion of cattle herds from the main livestock production areas of the country migrate as a coping mechanism to the ecological and environmental constraints. In particular, transhumance represents an integral component of the livestock production system in the Adamawa Region, with around 50% of the cattle herders implementing such a management practice [7, 8]. While herds in the North and Adamawa Regions usually migrate extensively covering long distances from their Region of origin, in the North-West and West Regions of the country most cattle herds tend to undertake a more local migration, largely within the Region [9]. However, knowledge of these migratory routes and trajectories in Central Cameroon is limited to anecdotal and informal reporting. Characterizing the seasonal transhumance trajectories and the nomadic herding patterns is, therefore, of importance for better understanding interactions within the livestock population and, hence, their potential implications for infectious diseases epidemiology and prevention.

Since 1997, several studies have investigated the movement behaviour of wildlife and livestock animals in Africa using global positioning system (GPS) technology [10–12] and, more recently, mobile phone systems [13]. Notably, GPS-tracking technology has been used in SSA to study grazing behaviour of free-ranging cattle and their response to the spatio-temporal variability of vegetation resources [14–21], to characterize the movements of nomadic pastoralist communities [22] and to collect data on the movements of both traders and traded herds [23]. However, to date, the formal application of GPS-tracking devices on transhumant cattle herds for the entire duration of the migration is still limited, and the understanding of transhumance routes, and associated migratory patterns, is particularly poor in the Central African region. Despite this paucity of specific information on the livestock transhumance patterns in Central Africa, previous investigations of grazing behavior of free-ranging cattle showed that the trajectory of a single animal is representative of the daily grazing orbit and movement patterns of the rest of the herd [16, 18, 21]. Tracking one animal from a migrating herd with GPS technology provides, therefore, a suitable framework for studying transhumance routes and migration patterns during long-distance movements.

Movements and contact patterns within and between animal populations are known to be central drivers of livestock disease dynamics [4] and empirical information, including precise seasonal cattle transhumance trajectories, would help informing a more evidence-based approach to animal health management in Cameroon. This builds on recent work for improving the understanding of cattle trade-related movements [24] and for identifying constraints for disease controls in pastoral and small-scale livestock husbandry and production systems in Cameroon [25]. An increased understanding of the common patterns and practices during this seasonal migration would help informing the veterinary authorities in designing interventions aiming at enhancing disease surveillance and improving disease control in the study areas.

Here, we present the first formal study of transhumance patterns in Cameroon, while assessing the feasibility of applying GPS collar devices on cattle herds for the entire duration of the migration. Cattle herds originally located in the Adamawa Region of Cameroon were tracked for a period of over six months. Upon their return from seasonal pastoral movements, a questionnaire-based survey was used to collect further information on tracked herds’ experience during their migration. The objectives of this study were (1) to characterise the seasonal transhumance routes and daily movement patterns of a restricted pool of cattle herds normally grazing in the Adamawa Region, and (2) to describe the main animal health related issues and interaction patterns during this long-distance migration.

## Methods

### Study area and herd identification

The Adamawa Region is mainly an open woodland Guinea savannah ecotype above 1000 m, covering an area of approximately 64,000 km2. It is considered to be the main cattle production area of Cameroon with a reported cattle population of about 1.25 million head of cattle [26].

Between October and November 2014, a convenience sample of ten cattle herds whose owner/herdsman were prepared to participate in the study were identified. It was possible to select herds originating/normally grazing in three different Divisions (one administrative level below Region) of the Adamawa Region. This convenience sample was the only applicable due to the nature of the context and the complexity of identifying suitable and available candidates willing to participate in the study. In similar settings, cattle herds have been observed to synchronise their behaviour, to a large extent, to more socially dominant individuals, particularly during travelling and grazing activities [27–29]. In each herd, one animal was selected to carry the GPS device. Selection was first based on discussions with the herdsman to identify socially dominant animals within their herd. Each identified animal was then inspected clinically to ensure that they were robust and healthy. Details on the animals chosen to carry the GPS device in each selected herd are shown in Table 1.

**Table 1:** Cattle herds identified in the Adamawa Region of Cameroon in October and November 2014

Herd/Collar number	Location (village of origin)	Administrative Division	Collar deployment date	Herd size	Tracked Animal and age (years)	Transhumance completed and survey carried out	Complete GPS data retrieved
1299	Likok	Vina	18/11/2014	45	cow (4y)	Yes	Yes
1300	Belel	Vina	25/10/2014	57	cow (4y)	Yes	Yes
1301	Nyambaka	Vina	24/10/2014	35	bull (4y)	Yes	Yes
1302	Likok	Vina	03/11/2014	40	bull (4y)	Yes	Yes
1303	Margol	Vina	03/11/2014	71	cow (5y)	Yes	No
1304	Mbe	Vina	03/11/2014	71	cow (4y)	No	No
1305	Dir	Mbere	05/11/2014	93	bull (4y)	Yes	Yes
1307	Lougga	Vina	19/11/2014	50	cow (6y)	Yes	No
1308	Martap	Vina	08/11/2014	45	cow (4y)	Yes	Yes
1350	Banyo	Mayo Banyo	30/11/2014	33	bull (4y)	Yes	No

Date of deployment of the GPS device depended on the date of start of the transhumance, the availability of the  herds' owners and locations of these herds. In brackets are reported the ages of the cattle (in year) that were selected to be tracked

### Data collection

Lightweight (320 g) GPS collars (Savannah Tracking Ltd., Kenya) with global system for mobile (GSM) communications network access and on-board backup data storage were fitted to each selected animal. The data collection schedule and data recording parameters were set through an on-line software interface. The GPS sampling frequency was set to every 2 h and transmission over the GSM network was set for once daily in accordance with other studies tracking cattle in similar settings [11, 15, 30]. More frequent GPS sampling would have provided the opportunity to increase the accuracy of the estimates of distances travelled between GPS locations over the observation period [31, 32]. However, the trade-off between data storage capacity, the expected duration of the transhumance and the GSM network coverage, led to identify this as the optimal sampling frequency for the objectives of the current study.

The GPS collars were retrieved upon return from transhumance in May 2015 and sent back to Savannah Tracking Ltd. in Kenya to retrieve the stored data that could not be downloaded through the GSM network. The lightweight (320 g) GPS collars were easily retrieved from the tracked animals of each herd that after the study continued to be part of their herds. The datasets from each collar unit were downloaded as csv files and included the complete record of the latitude and longitude and the travelling speed (km/hour) at each recording point and an estimation of the accuracy of the location. In addition, at the time of recovering the collar, a structured interview was carried out with the returning herdsmen. The questionnaire took 15–20 min to administer and aimed at collecting information on the daily routines of the herd and herd management practices during the transhumance period, including animal health conditions, interactions with livestock or wildlife populations and on trading activities. The hard copies of the questionnaires were manually transcribed to pre-designed Excel 2007 (Microsoft) spreadsheet and stored as a csv file.

### Data analysis

A combination of descriptive analytical approaches was applied to characterize the transhumance trajectories of the tracked cattle and to assess the general characteristics of the period of movements of the tracked herds. The recorded GPS coordinates and speed of travel of the tracked cattle were assessed using simple descriptive tools such as histograms and interquartile box plots.

The distance traveled between any two consecutive recorded GPS locations was estimated in kilometers using latitude/longitude (degree) georeferences and calculating the Euclidean distance between these GPS locations. The distances travelled were also aggregated at daily and weekly intervals in order to assess the variability of the distances travelled by the different tracked herds over different intervals. This was then used to characterize the range of daily distances traveled and to estimate the daily mean distances travelled during each of the weeks of observation.

A hot spot analysis [33] was carried out to assist identification of locations with unusual high concentrations of data points, identifying spatial clusters. In order to identify and count these hot spots, or activity locations, 2D kernel density were used [34]. Kernel density estimation is a non-parametric method where a symmetrical kernel function is superimposed over each GPS location and requires the definition of a spatial and temporal parameter [35]. The spatial parameter, or bandwidth value, corresponds to the roaming radius while the temporal parameter defines the minimal duration of stay at a given location to qualify as a significant stop. For the purpose of this study, the temporal parameter was given by the time between any two GPS recordings (2 h) and the spatial parameter (bandwidth) was set to a cell size likely to host all of the cattle of the tracked herd according to field observations (500 m) [34].

All analyses and graphics were performed using the raster [36], rgdal [37], ks [38] and ggplot2 [39] packages in the statistical software R version 3.2.3 [40].

### Ethical statement

This research was authorised by the Ministry of Livestock, Fisheries and Animal Industries (MINEPIA) (Research permit number: 0119/MINRESI/B00/C00/C010/nye), and approved by the Cameroon Academy of Sciences (approval number 0371/CAS/PR/ES/PO). In the United Kingdom approval was given by the Veterinary Ethical Review Committee (VERC) of the Royal (Dick) Veterinary School of the University of Edinburgh (approval number 28/14).

All methods were performed in accordance with the relevant guidelines and regulations and informed consent was obtained from all subjects. Interviewers were trained to provide the information regarding the consent process to be communicated to the participants and the informed consent was obtained from all subjects. Oral consent was obtained due to the variable level of literacy of the respondents. Prior to interviewing, the study objectives, procedures and the content of the questionnaires were also explained to the participants who were made aware that they were under no obligation to participate if they did not want to.

## Results

### Collar and data retrieval

Out of the 10 deployed GPS collar units, all of them were successfully retrieved from the animals in May 2015, including seven with complete records of spatial locations and three collars with partial records. Among the seven collars with complete records, one collar belonged to a herd whose herder finally decided not to go on transhumance. Partial recordings from the three collars were due to the memory being overwritten with later locations as a consequence of poor GSM network coverage which resulted in excessive use of the on-board memory storage. In one case (collar 1350) the recordings were almost entirely unavailable, likely due to a concomitant technical failure of the GPS device. Data retrieved from collars 1303 and 1307 were only partially complete, 42 and 44% of the transhumance days, respectively. As a consequence, these three collars were excluded and, along with the herd that failed to leave on transhumance, left data from 6 collars for analysis.

### Spatial movements of the tracked herds

**Fig. 1:**
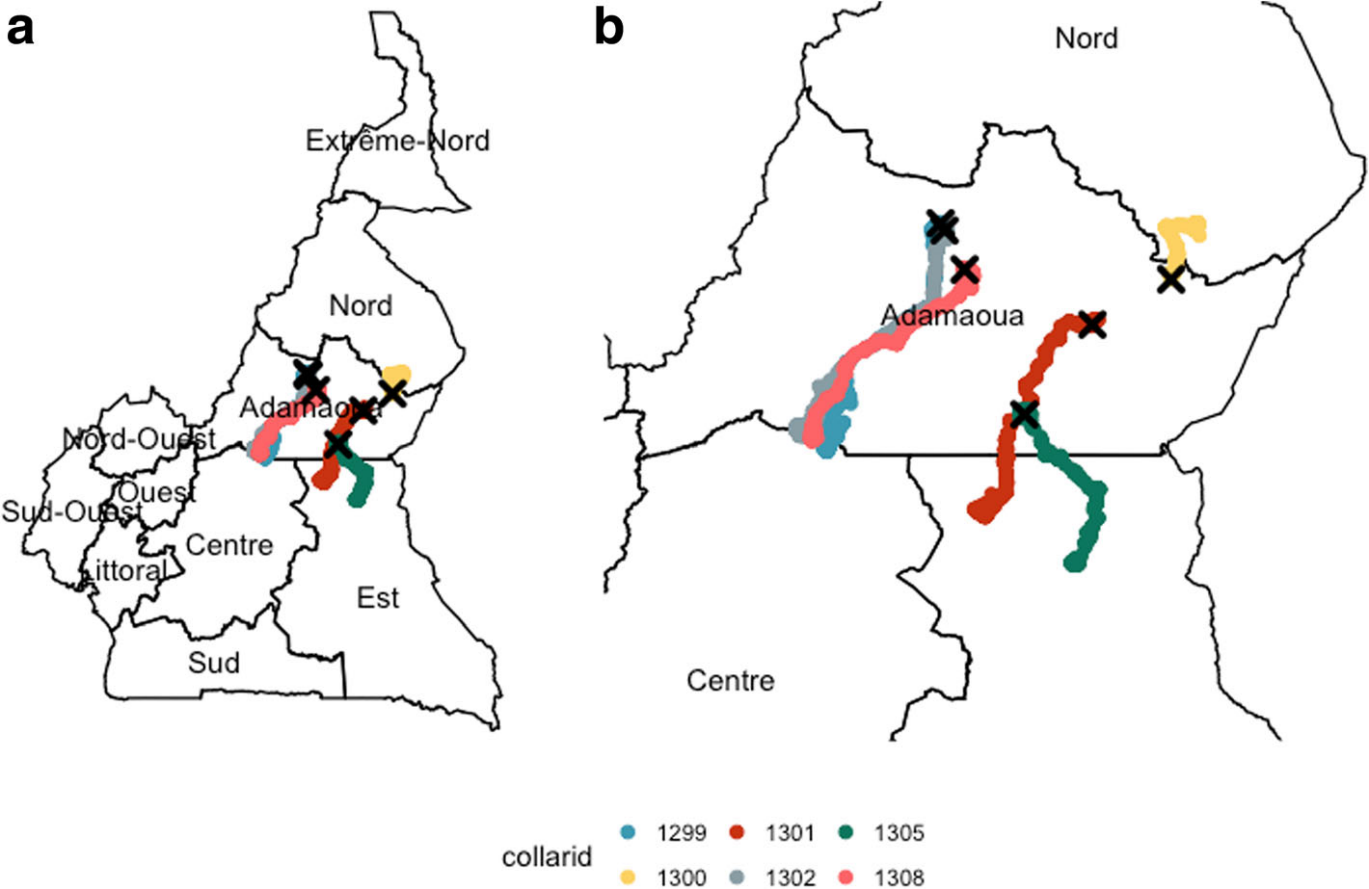
Transhumance trajectories of the 6 tracked herds that undertook seasonal migration in Central Cameroon (October 2014–May 2015) and that successfully recorded a full dataset. The trajectories of each GPS collar are displayed with a different colour on the section of the Cameroonian map. The black X indicates the starting point of the transhumance. Panel **a** displays the trajectories over the map of Cameroon, while Panel **b** focuses on the Regions of Central Cameroon

The six herds that went on transhumance showed different migratory patterns (Fig. 1 and Table 2). In most cases (5/6) the seasonal migration was towards the southern Regions of the country (herd 1299, 1302 and 1308 to the Centre Region; herd 1301 and 1305 to the East Region), while in one case it was towards the north, to the North Region (herd 1300). The three herds migrating to the Centre Region were directed towards areas of the Mbam and Djerem National Park (about 170 km of distance from their origin), while the two herds migrating to the East Region were directed towards the Pangar and Djerem Reserve (about 150 and 120 km from their origin, respectively). The herd migrating towards north was directed to the Mayo-Rey Division of the North Region of Cameroon, 53 km from its origin. The duration of the transhumance was relatively similar among the 6 herds and varied between 26 and 32 weeks. Although the straight line distances between the origin and the final destination of the transhumance ranged between 53 and 170 km, the overall estimated distance covered by the herds during the whole duration of the transhumance was relatively similar, ranging between 633 and 763 km.

**Table 2:** Distances travelled by the cattle herds during transhumance in Central Cameroon (October 2014–May 2015)

Herd/Collar number	Total distance covered (km)	Median distance per day (km)	Shortest distance between origin and destination (Km)	Transhumance duration (weeks)	Final num. Transhumance destination
1299	746	4.14 (SD 2.21)	170.9	26	Centre Region
1300	730	3.32 (SD1.74)	53.3	32	North Region
1301	763	3.97 (SD 2.03)	154.0	29	East Region
1302	633	3.23 ( SD2.01)	172.7	28	Centre Region
1305	649	3.55 (SD 1.91)	115.5	27	East Region
1308	726	3.63 (SD 1.56)	157.1	29	Centre Region

The total distance covered during the entire period of observation, the median distance travelled per day (standard deviation in brackets) and the distance between the two most far apart locations are all reported in kilometers. The duration of the transhumance, in weeks, is estimated from the week the herds left the grazing location in the Adamawa Region until the week they returned to same location

### Speed and daily movements

**Fig. 2:**
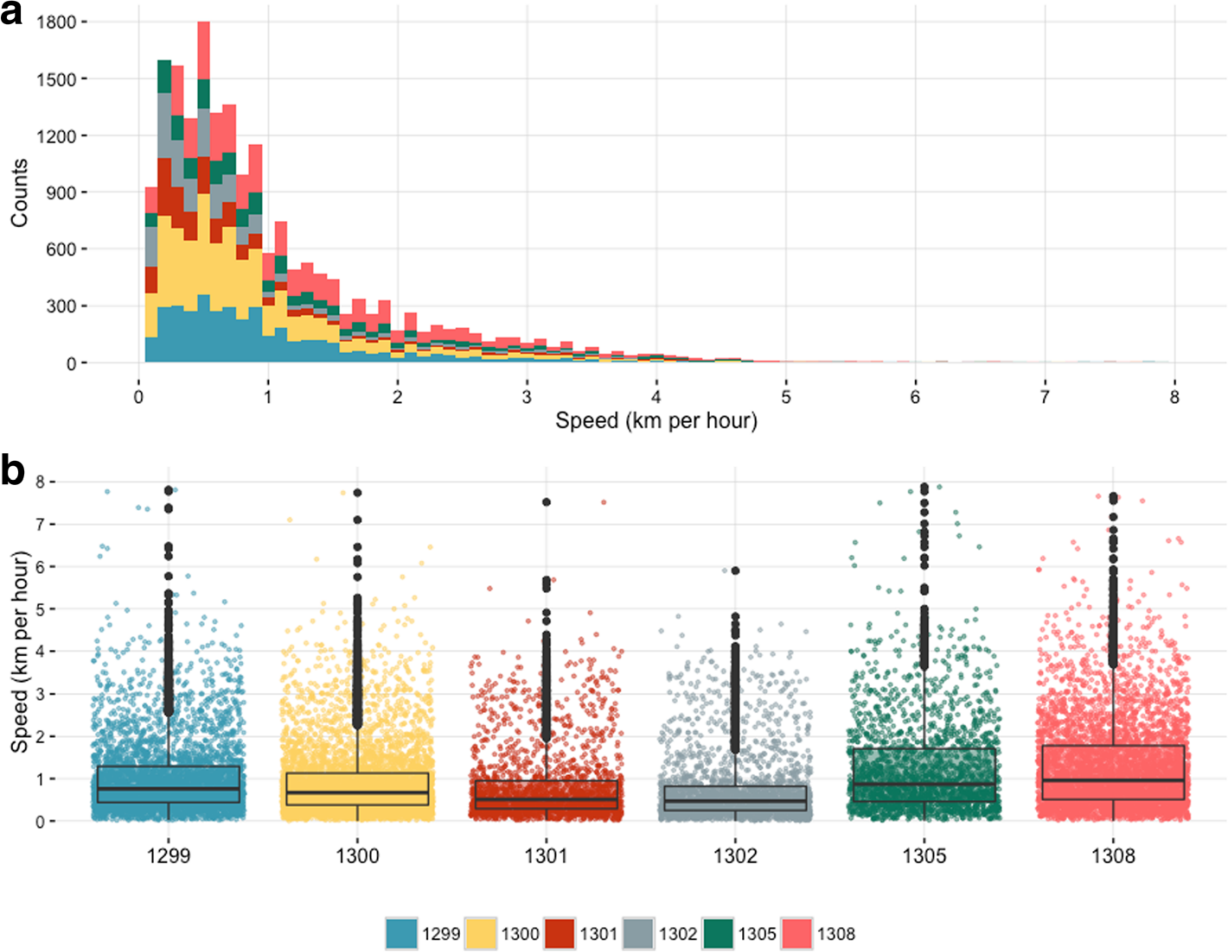
Speed of movement of the transhumant herds tracked between October 2014 and May 2015 in Central Cameroon (in km/hour). **a**: Distribution of the recorded speed of movements at each GPS captured location for the tracked cattle (km/hour on the x-axis and counts on the y-axis). **b**: Boxplot of the recorded speed of movements at each GPS captured location for each tracked cattle (x axis). For each box the dots refer to the recorded speed at each GPS captured location, the upper and lower hinges correspond to the 1st and 3rd quartiles (the 25th and 75th percentiles) and the horizontal line to the median value

The speed of movement of the tracked herds, as recorded every 2 h, ranged between 0.1 and 7.8 km/hour (Fig. 2a). The overall median speed of each of the 6 herds during the whole period of observation ranged between 0.48 and 1.02 km/hour (Fig. 2b). Although the absolute range of the recorded speed was approximately similar between the herds, herds 1305 and 1308 displayed a wider interquartile range of speeds compared to the other herds (0.48–1.82 km/hour and 0.51–1.91 km/hour, respectively) (Fig. 2b).

Overall, herds showed consistent patterns of movements across the 24 h cycle. The mean speed of the herds tended to increase between 06:00 and 18:00 h. Similarly, the absolute peaks of speed were recorded within this time window (Additional file 1: Figure S1). Nevertheless, across the whole study period, all the 6 herds were recorded to have moved at least 4 km/hour, during all of the recorded time points throughout the 24 h daily cycle. In other words, during the study period herds were recorded making significant movements even during the night.

**Fig. 3:**
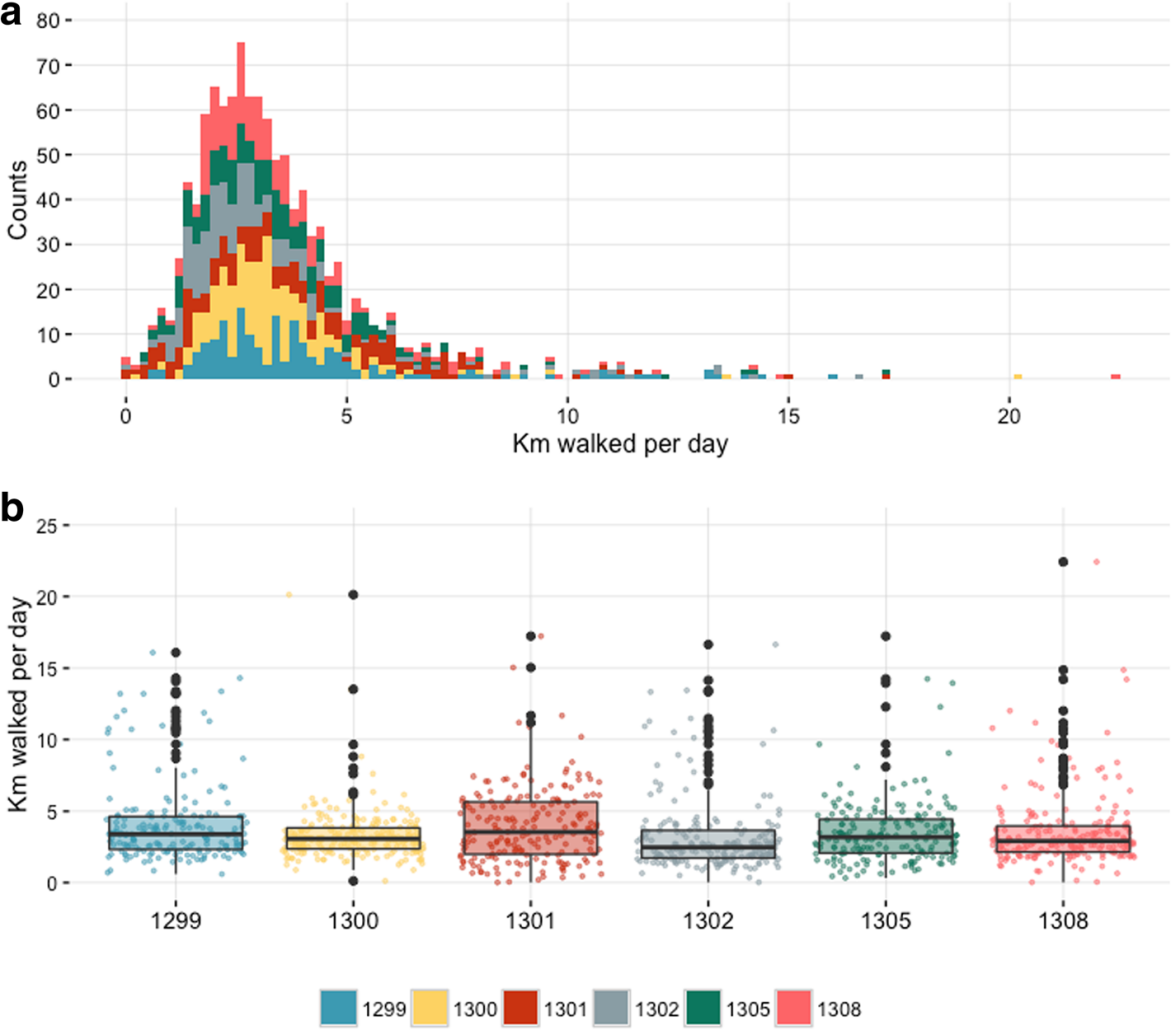
Daily distances covered by the transhumant herds in Central Cameroon between October 2014 and May 2015. **a**: Distribution of the daily distances walked by the tracked cattle (distance in km on the x-axis and counts on the y-axis). **b**: Boxplot of the daily distance travelled by each tracked cattle (x-axis). For each box the dots refer to the the daily distance for each day of observation, the upper and lower hinges correspond to the 1st and 3rd quartiles (the 25th and 75th percentiles) and the horizontal line to the median value

**Fig. 4:**
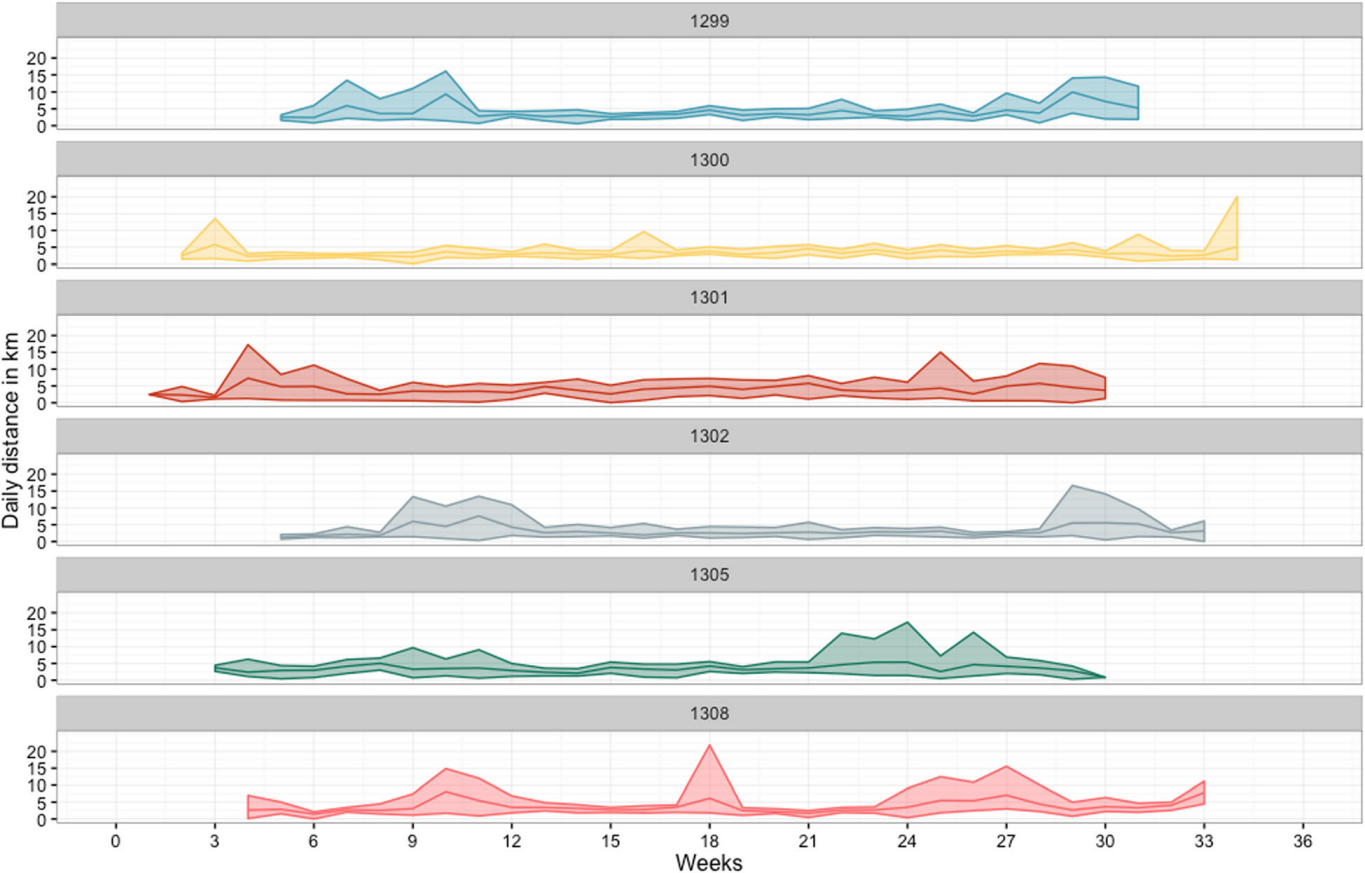
Mean and ranges of the daily distances walked during each week of the transhumance period between October 2014 and May 2015 in Central Cameroon. For each tracked cattle the mean estimated daily distance walked during each week of observation is represented by the middle line while the upper and lower lines represent, respectively, the largest and shortest distances walked in each weeks

The daily distance travelled by a herd ranged between 0.3 and 22.9 km/day, with 86% of herd-days below 5 km/day (Fig. 3a). The median distance covered per day by each herd over the whole transhumance period ranged between 3.2 and 4.1 km/day (Fig. 3b). Only during relative short periods greater daily distances were travelled and these were mainly at the start and end of the migration, reflecting movements from and to the main transhumance grazing zone (Fig. 4). However, in 3 cases (herds 1299, 1305 and 1308) greater daily distances were also travelled during other weeks of the transhumance. Overall, these periods of higher median daily travelled distance tended to last between 1 and 3 weeks (Fig. 4). Herd 1300 was an exception, with an overall shorter weekly median daily distance travelled across the whole period of observation.

**Fig. 5:**
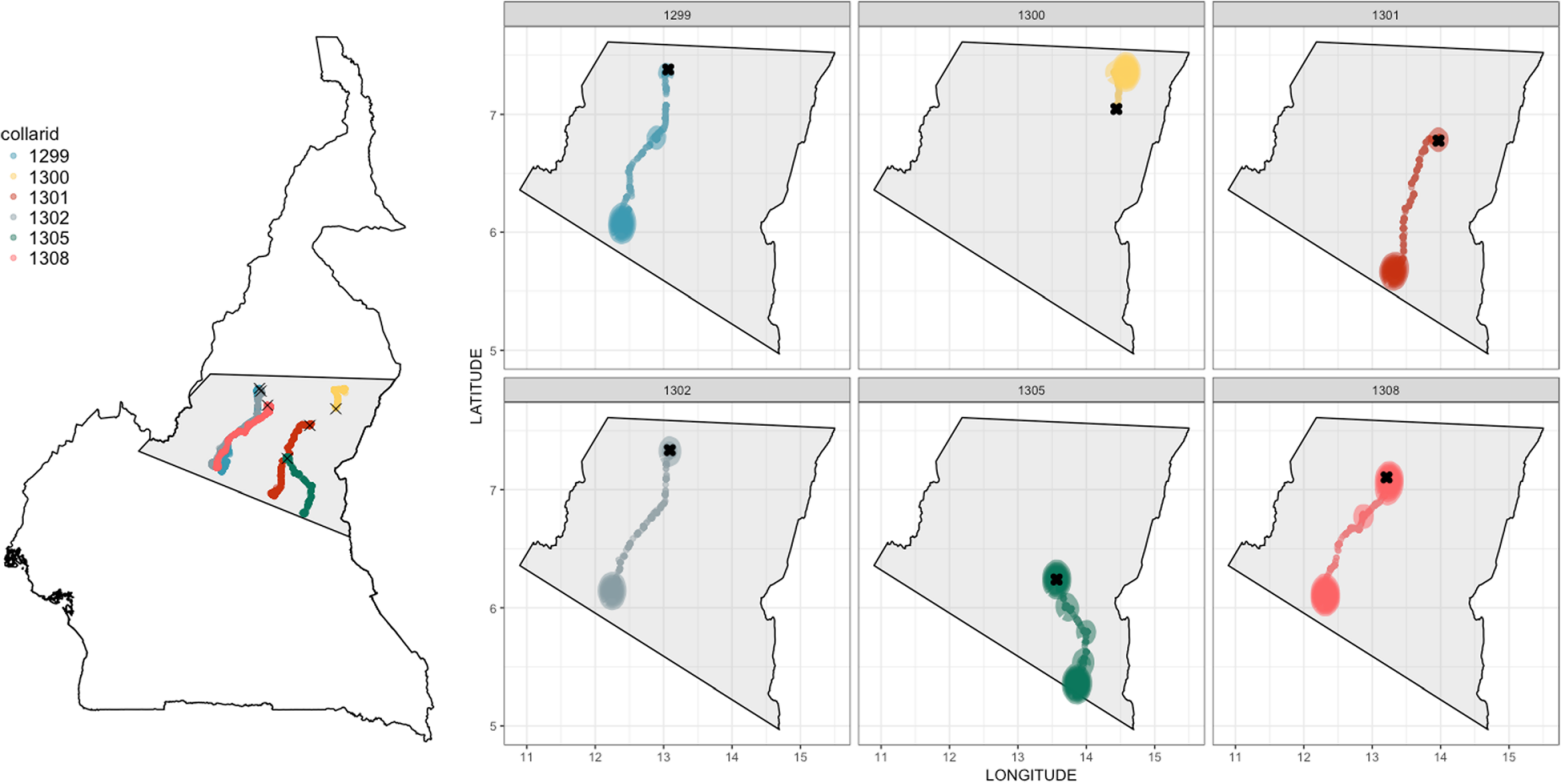
Hot spot analysis of the locations visited during the transhumance period between October 2014 and May 2015 in Central Cameroon. On the left side of the figure the trajectories of each GPS collar are displayed with a different colour on the section of the Cameroonian map. On the right side two-dimensional density plots of the recorded GPS locations. The trajectories are displayed by the dots of different colour representing the GPS locations recorded per each collar. The intensity of the colour reflects the occurrence (count) of GPS recordings (counts of observations) at each specific area (e.g.: most of the GPS locations of collars number 1301 and 1302 were recorded in two locations while, on the contrary, the GPS locations of collar 1305 were mainly recorded in 5 areas). In both sides of the figure the black X indicates the starting point of the transhumance

The hot spot, or activity locations, analysis, also showed geographical areas where the herds spent longer periods of activity and areas where, by the contrary, the herds were only transiting (Fig. 5). For all of the tracked herds the origin and destination of the migration represented hot spots of activity. Nevertheless, herds 1299, 1305 and 1308 spent longer periods also in other zones along their transhumance routes (Fig. 5).

### Contacts and interactions with other cattle herds during transhumance

**Fig. 6:**
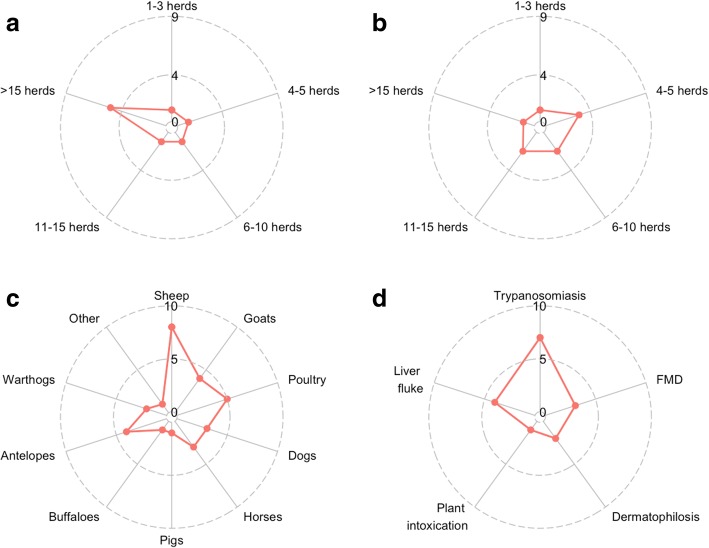
Encounters with other animal species and common health problems reported during transhumance in Central Cameroon (October 2014–May 2015). **a** and **b** report the frequency of specific answers from each of the 9 interviewees. **c** and **d** report the sum of the times animals species and diseases were mentioned by the 9 interviewees. **a**: Reported number of cattle herds encountered on average every day during the trekking towards transhumance destinations or returning towards the usual grazing locations. The plot displays the answers of the interviewees and the red line refers to the number of reports by the 9 interviewees. **b**: Reported number of cattle herds encountered on average every day during grazing activities at the transhumance destination. The plot displays the answers of the interviewees and the red line refers to the number of reports by the 9 interviewees. **c**: Reported species of domestic and wild animals encountered during the transhumance. The plot displays the animal species and the red line refers to the number of reports by the interviewees. **d**: Reported health problems faced by the herds during the transhumance. The plot displays the reported health problems and the red line refers to the number of reports by the interviewees

During periods of active trekking and movement towards transhumance destinations or returning back from these locations, 5/9 herdsmen reported that > 15 cattle herds were usually encountered each day (Fig. 6a). The four other herdsmen reported routinely encountering 1–3, 4–5, 6–10 and 11–15 other cattle herds per day, respectively. In contrast, in grazing areas such as the transhumance destinations, herds tended to meet fewer other herds per day: only one herdsman reported that more than 15 cattle herds were encountered on an average day at this location, 2 herdsmen reported a mean of 11 to 15 and all the other reported fewer contacts (Fig. 6b).

The typical duration of these encounters was estimated by 6/9 herdsmen to last less than 1 h, while 2/9 herdsmen reported a duration of interaction between 4 and 6 h and 1/9 between 13 and 24 h (Additional file 1: Figure S2). Interestingly, two of the tracked herds (1301 and 1305) physically met during the period of observation (Additional file 1: Figure S3). The encounter lasted, approximately, between 4 and 6 h, while herds were returning from the seasonal migration, and was in a usual grazing location for the herd 1305, but about 59 km from the migration origin of herd 1301.

### Contacts with other animal species and health issues reported during transhumance

Herders were asked what other animal species had been encountered during the transhumance. Among domestic species, sheep were the most frequently encountered and reported by all the 9 interviewees, followed by poultry (5/9 herdsmen), goats (4/9 herdsmen), horses and dogs (3/9 herdsmen) and pigs (1/9 herdsmen). The most frequently encountered wildlife species were the antelopes (4/9 herdsmen), followed by warthogs (2/9 herdsmen) and buffaloes (1/9 herdsmen) and another unspecified animal (1/9 herdsmen) (Fig. 6c).

Among the reported health problems faced by the cattle herds during the transhu- mance period, the most commonly reported was trypanosomiasis (7/9 herdsmen) followed by liver fluke (4/9 herdsmen), foot and mouth disease (FMD) (3/9 herdsmen), dermatophilosis (2/9 herdsmen) and plant intoxication (1/9 herdsmen) (Fig. 6d).

When assessing the causes of death of cattle during transhumance, accidents were reported as having caused the loss of at least one animal (5/9 herdsmen), followed by disease (4/9 herdsmen) and by plant intoxication (3/9 herdsmen) (Table 3).

**Table 3:** Number of herdsmen reporting cattle death and trade during the transhumance period (October 2014–May 2015). NH: Number of herdsmen reporting events; NC: Number of cattle involved in each reported event

Reported events		N_H_	N_C_
Causes of cattle losses	Accident	5	7
	Diseases	4	6
	Plant Intoxication	3	10
Cattle trade	Sale (within market)	4	13
	Sale (outside market)	2	4
	Purchase (within market)	0	0
	Purchase (outside market)	0	0

Reported cattle losses during transhumance were stratified by the cause of death as diagnosed by herdsmen. Reported trade events during transhumance were stratified by whether animals were sold (or purchased) within or outside the trading system (livestock markets)

### Trading activities during transhumance

None of the herdsmen (0/9) reported having acquired cattle during the transhumance period, either from livestock markets or from outside the trading system. However, four (4/9) herdsmen reported having sold cattle at livestock markets during the transhumance, while two (2/9) herdsmen reported having sold cattle outside the trading system (Table 3). When traded at the market place, respectively, 2/45, 3/35, 3/40 and 5/93 cattle were sold, while 1/45 and 3/57 cattle were sold outside the market place (Table 3).

## Discussion

In SSA, seasonal livestock mobility is an important adaptation mechanism for pastoralist communities, and a key strategy to manage the variability of the natural resources in the ecosystem [1]. In Cameroon, transhumance is a common practice for many pastoralists and their cattle herds to cope with the ecological and environmental constraints of the dry season. However, knowledge of migratory routes and patterns in Central Cameroon, and their potential implications for infectious diseases epidemiology and prevention, is still limited. Here, we characterized migrating patterns of a few, but representative, GPS-tracked cattle herds and described key activities and experiences along their transhumance across Central Cameroon.

Long daily distances were relatively rarely traveled (approximately 15% of the recorded days), typically at the beginning and the end of the transhumance. During these periods of more active mobility, an average speed of movement compatible with traveling behavior (between 3 and 4 km/hour) was mostly recorded during the daylight, consistently to reports in the East African rangelands [41]. However, herds were traveling at any time during transhumance, irrespective of being day or night. Overall, the proportion of daily distances traveled and the variability of walking speed are in line with previous findings in East and West Africa [31, 41, 42].

Over the six GPS-tracked herds we found three cattle herds having multiple repeated traveling and grazing periods through different temporary transhumance locations. Similarly to previous findings in SSA [41, 43], hence, the seasonal cattle transhumance in Central Cameroon, rather than a simple transit between two locations, tended to be a more complex journey through multiple grazing areas. This migration lasted a significant length of time (even greater than half of the year). Furthermore, the trajectories of all of the three tracked herds moving from the Central-Eastern part of the Adamawa Region towards the Centre Region highlighted the presence of a common migratory route, or transhumance corridor. In Northern Cameroon, and the larger Chad Basin, pastoralists and their herds move through established transhumance corridors connecting seasonal grazing lands [44]. Further confirmation of the observed migratory routes between the Adamawa and the Central Regions can provide evidence of common transhumance corridors and important indications for designing strategic and efficient veterinary interventions. For example, surveillance posts could be established along these corridors for providing animal health services to the migrating herds (e.g. free dipping or spraying points), and potentially using these locations for control measures (e.g. vaccination points). In addition, if the common transhumance destinations in the Central Region can be further confirmed for a high number of cattle herds, and over multiple years, it would suggest these zones should be considered as high cattle density areas in Cameroon, at least for a significant part of the year. This information would provide evidence for appropriately considering these areas within infectious diseases surveillance and control strategies.

Multiple grazing locations during transhumance increase exposure of herds to geographically limited or seasonally abundant diseases [5]. In our survey, most herdsmen reported trypanosomiasis and liver fluke as health issues for their herds. This finding suggests that, while grazing areas provide greener pastures and greater water resources (e.g. natural water points) for transhumant herds, they also offer ideal habitats for vector and parasites proliferation [45]. As such, transhumant herds potentially contribute to the persistence and circulation of vector-borne diseases and parasites in Cameroon [45].

Because of the complexity of engaging stakeholders in this study, it was not possible to use a statistically robust sampling approach. Instead, we used a convenient sampling of a limited number of cattle herds. Clearly, this is a major shortcoming of this survey and additional studies, with increased number of herds, are required to confirm our findings. In particular, our survey provides information of only a few diseases affecting cattle herds through anecdotal reporting and without any supporting laboratory evidence. Although this list is certainly not exhaustive, we believe that it represents the list of infectious diseases which are perceived by herdsmen as the most important for their herds. It is also worth noting that the reported health conditions included infectious diseases for which direct contacts between animals are key transmission mechanisms (e.g. FMD and dermatophilosis), and which are well recognized by livestock stakeholders in the study areas [46].

During transhumance, particularly while traveling towards grazing areas, cattle herds tended to have more frequent contacts with other herds and with wildlife, compared to when they are sedentary at grazing locations. Despite this variability in contact rates, the interactions between cattle herds, both during traveling and at grazing areas, were reported to have relatively short durations (< 1 h). Nevertheless, a short contact time, particularly if at close proximity, could be sufficient for the transmission of highly infectious diseases, especially during the peak of the infectious periods [47, 48].

Over the eight months of study, two tracked herds, which originated from very distant areas, were recorded in contact at the same grazing location for 4 to 6 h. This observation confirms that opportunities for close interactions occur not just locally, at transhumance destinations with communal grazing, but also through the transhumance migration routes. This finding further reinforces the potential strategic role of veterinary surveillance and control points along the migratory routes, or transhumance corridors. Veterinary check points would represent key locations for designing and implementing efficient surveillance and control measures against infectious and parasitic diseases, including other priority livestock diseases in Cameroon other than the ones highlighted in this study, such as pasteurellosis, Contagious Bovine Pleuropneumonia (CBPP) and tick-borne diseases [45].

During the transhumance period, herdsmen of six of the nine herds under study reported to have sold cattle, either within or outside the formal trading system. Although livestock markets are known to greatly influence the spread of multiple infectious diseases throughout livestock industries [49–51], they are also places where social and cultural interactions occur. Such a report highlights the role of markets as an interface between the pastoral and the trading systems in the country. It also underlines their potential complementary role for risk-based approaches to surveillance, control and communication strategies for pastoral communities.

Although infectious diseases are the major animal health problem for the livestock sector in Cameroon [3, 45], the most commonly reported causes of death for cattle during transhumance included accidents and plant intoxications. Despite the small sample size available for this study, these findings show that transhumance presents specific challenges. Trekking for long distances poses specific risks and higher exposures for various types of physical accidents and environmental hazards, including plant intoxication for which very little knowledge is currently available. Furthermore, long distance livestock migrations may generate conflicts between pastoralists and local farming communities over limited natural resources and damages to crops [52, 53].

The potential tension then arising could pose additional security challenges to herds, as informally reported during the data collection of the current study.

## Conclusion

This limited study provides a general characterization of cattle transhumance pat- terns and of the key associated issues in Central Cameroon. The spatial and temporal overlapping between tracked cattle herds highlighted the opportunity for direct contacts and interactions between herds of distant origins, as well as with other domestic and wildlife species, both during traveling and grazing periods. The recorded speed of movement and interaction frequencies, and durations, hence, could potentially inform parametrization of further epidemiological studies.

Specific infectious and parasitic diseases were reported affecting the migrating herds, however physical accidents and environmental hazards (e.g. plant intoxications) were also reported as key factors impacting these herds. Importantly, the transhumant herds have also been shown to connect to the formal cattle trading system, highlighting the complexity of the pastoral, but increasingly market-orientated, livestock system in the country.

The overall characterization of transhumnce patterns in the study areas, and the related key aspects, represent a preliminary step for better understanding their implications in the epidemiology of livestock infectious diseases, and for their potential applications to inform surveillance and control strategies.

The further confirmation of some of the characterized migratory routes as common transhumance corridors, would provide the evidence to strategically design robust and efficient surveillance and control interventions at key locations.

Increased knowledge and understanding of pastoral movements and contacts between livestock populations at local and long-distance levels is essential for supporting the veterinary services in designing and planning more effective surveillance and control strategies of infectious diseases. Considering the key role of livestock transhumance as a key adaptation and ecological management mechanism for pastoralist communities in the region, this seasonal migration should be increasingly addressed in animal health management.

## Additional file

Additional file 1: **Figure S1.** Mean and ranges of the speed over the 24 h period. For each tracked cattle the speed of movements was recorded every two hours of the observation period. The middle line represents the mean speed at that hour of the day while the upper and lower lines represent, respectively, the fastest and slowest speed recorded at that specific time during the observation period. **Figure S2.** Reported duration of interaction with other cattle herds. On the x axis the reported usual duration of interaction with other cattle herds during transhumance and on the y axis the number of interviewees. **Figure S3.** Recorded encounter between 2 tracked herds. The herdsmen of these 2 herds (1301 and 1305) reported having met each other at the time of interview. The analysis of the GPS recordings enabled to identify the exact time and location of this encounter. The herds were recorded interacting for about 4 h between 8 am and 12 am of the 23rd April 2015, while returning to their respective grazing locations for the rainy season. (PDF 710 kb)

## Abbreviations

CBPP: Contagious Bovine Pleuropneumonia
FMD: Foot and mouth disease
GPS: Global positioning system
GSM: Global system for mobile
MINEPIA: Ministry of Livestock, Fisheries and Animal Industries
SSA: Sub-Saharan Africa
