# Supplementary material for: Cattle transhumance and agropastoral nomadic herding practices in Central Cameroon
Source: BMC Vet Res. 2018 Jul 3;14:214. doi: 10.1186/s12917-018-1515-z (PMC6029425; doi:10.1186/s12917-018-1515-z)
Supplement: Supplementary file 1 — Figure S1. Mean and ranges of the speed over the 24 h period. For each tracked cattle the speed of movements was recorded every two hours of the observation period. The middle line represents the mean speed at that hour of the day while the upper and lower lines represent, respectively, the fastest and slowest speed recorded at that specific time during the observation period. Figure S2. Reported duration of interaction with other cattle herds. On the x axis the reported usual duration of interaction with other cattle herds during transhumance and on the y axis the number of interviewees. Figure S3. Recorded encounter between 2 tracked herds. The herdsmen of these 2 herds (1301 and 1305) reported having met each other at the time of interview. The analysis of the GPS recordings enabled to identify the exact time and location of this encounter. The herds were recorded interacting for about 4 h between 8 am and 12 am of the 23rd April 2015, while returning to their respective grazing locations for the rainy season. (PDF 710 kb) [file 12917_2018_1515_MOESM1_ESM.pdf]

Supplementary Material: Cattle transhumance and agropastoral nomadic herding practices in Central Cameroon

Paolo Motta 1* , Thibaud Porphyre 1 , Saidou M. Hamman 2 , Kenton L. Morgan3 , Victor Ngu Ngwa4 , Vincent N. Tanya 5 , Eran Raizman 6 , Ian G. Handel 7 and B. Mark de C. Bronsvoort 1

*Correspondence: Paolo Motta Paolo.Motta@roslin.ed.ac.uk 1

SUPPLEMENTARY FIGURES

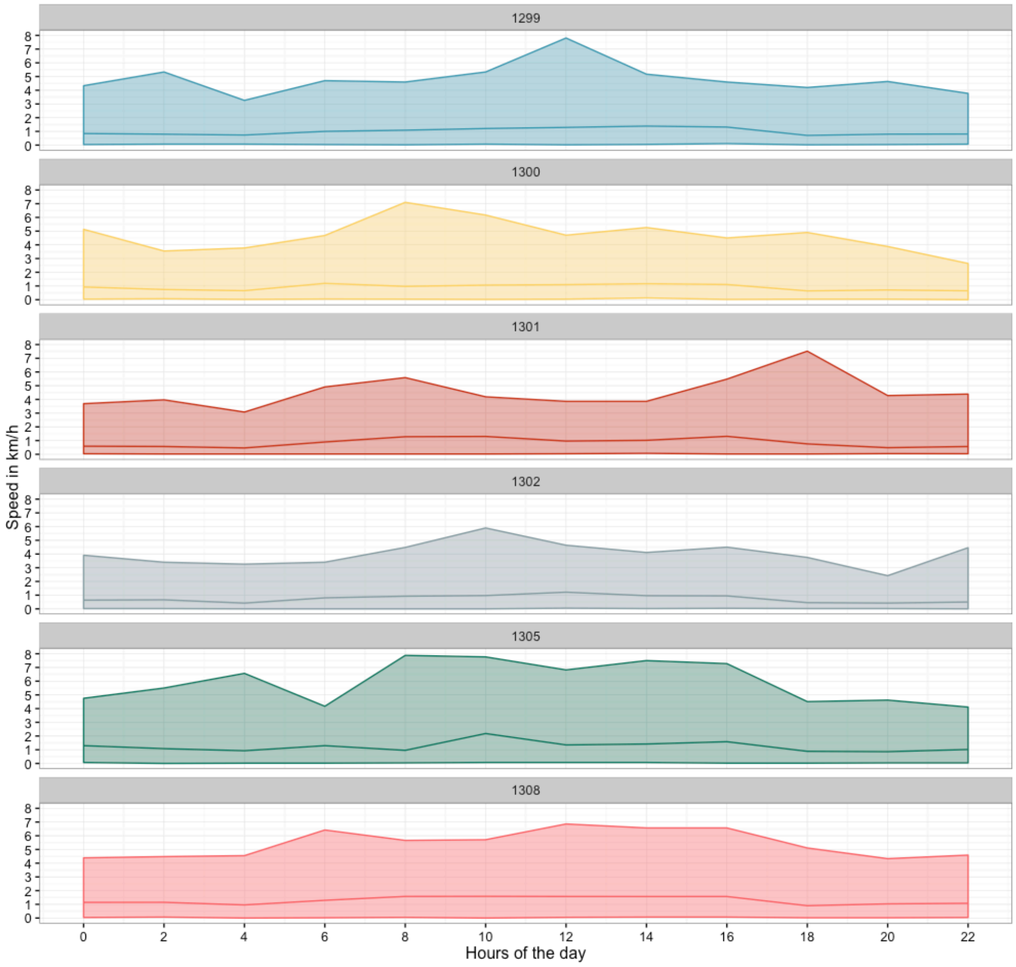

Figure S1. Mean and ranges of the speed over the 24 hours period. For each tracked cattle the speed of movements was recorded every two hours of the observation period. The middle line represents the mean speed at that hour of the day while the upper and lower lines represent, respectively, the fastest and slowest speed recorded at that specific time during the observation period.

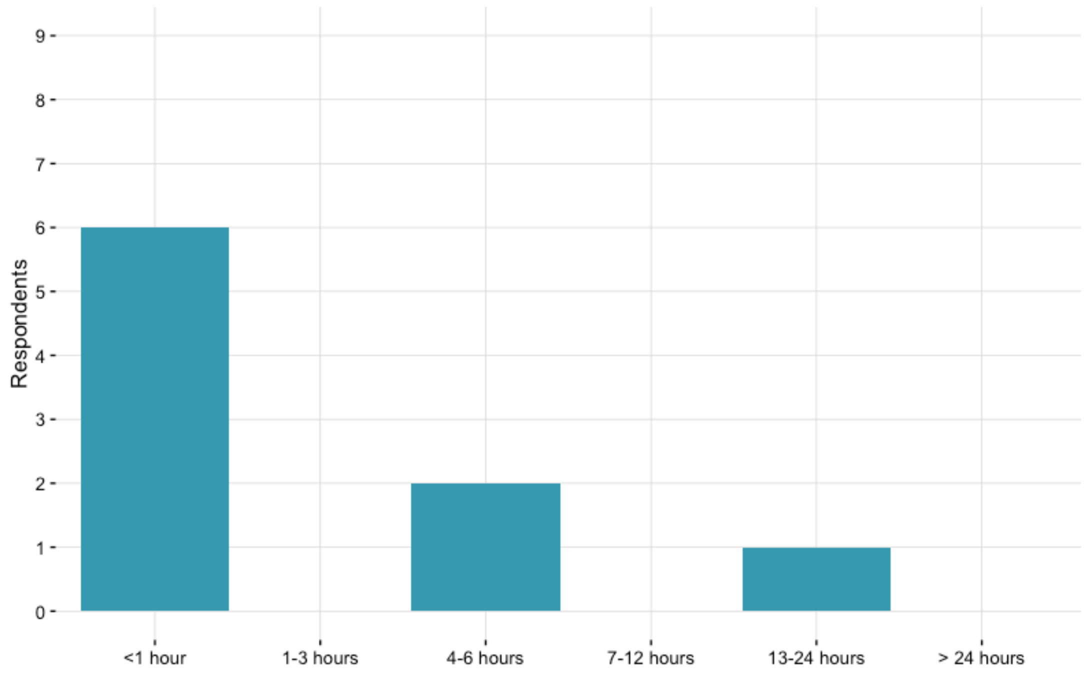

Figure S2. Reported duration of interaction with other cattle herds. On the x axis the reported usual duration of interaction with other cattle herds during transhumance and on the y axis the number of interviewees.

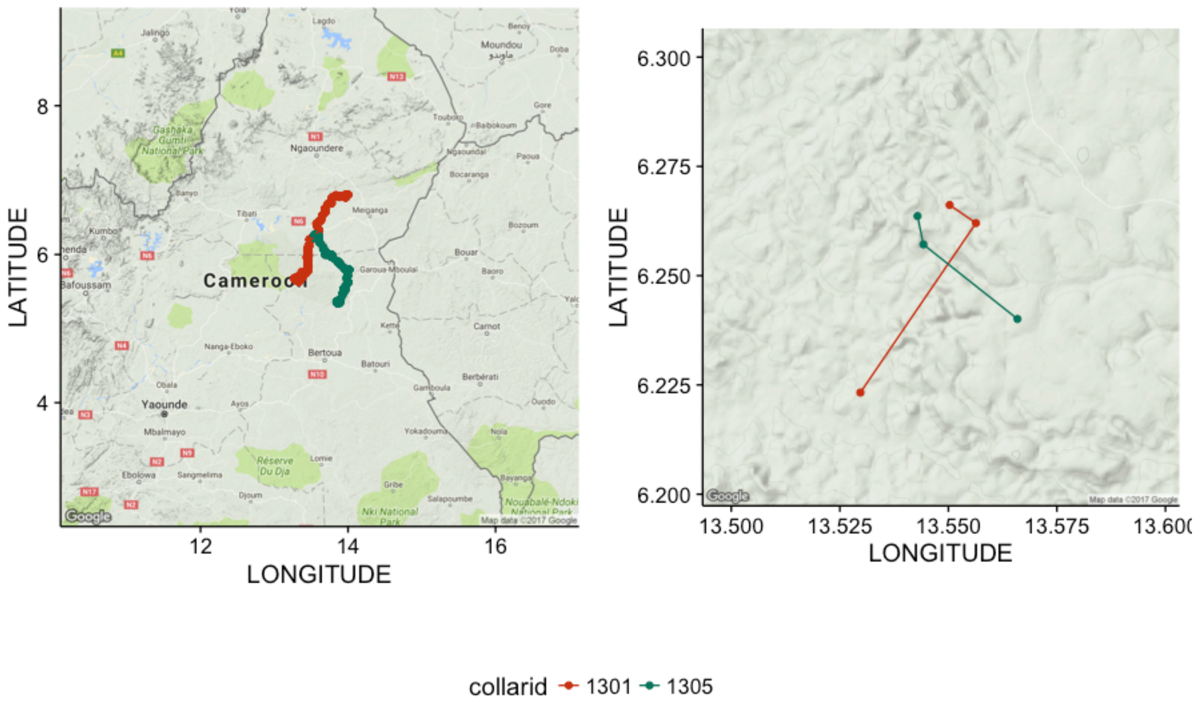

Figure S3. Recorded encounter between 2 tracked herds. The herdsmen of these 2 herds (1301 and 1305) reported having met each other at the time of interview. The analysis of the GPS recordings enabled to identify the exact time and location of this encounter. The herds were recorded interacting for about 4 hours between 8am and 12am of the 23rd April 2015, while returning to their respective grazing locations for the rainy season.
